# Prevailing over Adversity: Factors Counteracting the Long-Term Negative Health Influences of Social and Material Disadvantages in Youth

**DOI:** 10.3390/ijerph15091842

**Published:** 2018-08-27

**Authors:** Ylva B. Almquist, Evelina Landstedt, Josephine Jackisch, Kristiina Rajaleid, Hugo Westerlund, Anne Hammarström

**Affiliations:** 1 Department of Public Health Sciences, Centre for Health Equity Studies (CHESS), Stockholm University, SE-106 91 Stockholm, Sweden; josephine.jackisch@su.se; 2 Department of Public Health and Clinical Medicine, Epidemiology and Global Health, Umeå University, Norrland University Hospital, SE-901 85 Umeå, Sweden; evelina.landstedt@umu.se; 3 Stress Research Institute, Stockholm University, SE-106 91 Stockholm, Sweden; kristiina.rajaleid@su.se (K.R.); hugo.westerlund@su.se (H.W.); 4 Department of Public Health and Caring Sciences, Uppsala University, SE-751 05 Uppsala, Sweden; anne.hammarstrom@pubcare.uu.se

**Keywords:** disadvantages, living conditions, longitudinal, resilience, self-rated health, youth

## Abstract

Disadvantaged circumstances in youth tend to translate into poor health development. However, the fact that this is not always the case has been seen as indicative of differential resilience. The current study highlights factors outside the context of the family with the potential to counteract the long-term negative influences of social and material adversity in adolescence on general health status. This study was based on two waves of questionnaire data from the Northern Swedish Cohort. From the wave in 1981 (age 16), indicators of social and material conditions as well as factors related to school, peers, and spare time were derived. From the wave in 2008 (age 43), information about self-rated health was used. Ordinal logistic regression models (*n* = 908) showed that adversity in youth was associated with poorer self-rated health in midlife among men and women alike, net of health status at baseline. However, having an advantaged situation with regard to school, peers, or spare time appeared to protect against the detrimental influences of disadvantaged circumstances in the family context on subsequent health. This suggests that health-promoting interventions may benefit from focusing on contexts outside the family in their effort to strengthen processes of resilience among disadvantaged youths.

## 1. Introduction

Childhood is pivotal to life chances, including adult health, and the empirical research linking early experiences of adversity to later health outcomes has proliferated over the past decade [1,2,3,4,5,6,7,8]. Generally, two types of environmental exposures have dominated the literature on social determinants of health: social factors and material factors. Such factors reflect general poor living conditions and lack of resources, including overcrowding, financial difficulties, and limited social networks. In light of these findings, it is perhaps easy to forget that many individuals who grow up under adverse circumstances fare rather well. Like dandelions that are capable of surviving under almost any conditions, and even sprout through concrete, human beings have extraordinary capacity to adapt to their circumstances [9]. The concept of resilience has been used to describe such situations, where “a child prevails over adversity” [10]. However, despite a longstanding scientific interest in resilience, there have been few large-scale longitudinal studies that have included both men and women from the general population in their samples, used multiple indicators of adaptation, followed the individuals across a sufficiently long period of their lives, and kept the attrition rates low [11]. Moreover, according to a recent systematic review of research on amenable resilience factors that could influence the relationship between childhood adversities and later well-being, a strong dominance of individual/family-level psychological factors can be noted [12]. Consequently, there is a lack of studies addressing protective factors present in the broader socioecological system of the individual [13]—so-called community resilience factors—that may be more easily modified by public policy.

In order to address the above-mentioned gaps in the literature, the current study aims to examine multiple factors related to the school, peers, and spare time and whether these factors may buffer against the possible long-term negative health influences stemming from social and material adversity in youth. The analyses are based on a cohort study of around 1000 boys and girls of 16 years of age who were living in a Northern Swedish town in 1981, and they were followed up until their early 40s, with almost no loss to follow-up.

### 1.1. Childhood Adversity and Adult Health—Accumulation of (Dis)Advantages

Different models have been proposed to explain how and under what conditions childhood disadvantage may influence adult health. We take our point-of-departure in the theory of cumulative advantage/disadvantage [14]. Focusing on disadvantage, the overall idea behind this theory is that any specific disadvantage early in an individual’s life tends to be part of a broader clustering of adverse exposures that accumulates as the child approaches young adulthood and continues to gradually worsen across the life course. As such, it does not only illustrate how individual lives develop with increasing age, but how a cohort becomes differentiated over time, thereby contributing to the overall patterns of inequality at the societal level [15]. These ideas have been further developed into a framework called cumulative inequality theory, paying more attention to the mechanisms that underlie processes of cumulative (dis)advantage—or, how inequalities may “get under the skin” [16]. Cumulative inequality theory builds on the statement that inequalities are generated by social systems and manifested over individuals’ life courses through demographic and developmental processes. Even before a child comes into the world, her conception, foetal developmental, and birth is shaped by social forces. Moreover, childhood conditions are important for explaining how adult living conditions are shaped. This is particularly the case for childhood conditions related to the family lineage, in terms of both genetic transmission and the shared environment. Another key argument of cumulative inequality theory is that disadvantages increase the exposure to risk, whereas advantages increase the exposure to opportunity. However, cumulative inequality theory argues that life course trajectories are shaped not only by how risk and available resources accumulate, but also by human agency. Trajectories can thus be modified depending on how the individual’s respond to exposures to risk and opportunity. The resources available to the individual, both internal (e.g., coping strategies) and external (e.g., social support), influence this response and create “turning points”. This idea bears a lot resemblance to the notion of resilience, which will be discussed in more detail below.

### 1.2. A Resilience Perspective

The notion of dandelion children is largely what has guided the inquiry into resilience, from its place within the larger field of developmental psychology, over the past 40 years. Examples of pioneering research into resilience encompass Norman Garmezy’s studies of “atypical” patterns among youth at risk of psychopathology, the seminal work by Emmy Werner on protective factors among infants born in the Hawaiian island of Kauai, and Michael Rutter’s clarification of processes behind resilience in terms of risk effect reduction [16]. While resilience was initially thought of as a personality trait that would enable the individual’s use of internal resources to adjust to stressors and stress, this view has gradually shifted to encompass developmental processes as related to external resources. The focus on resilience as interacting person–environment systems [9] has much in common with human ecological theory [13]. More specifically, it involves the recognition that resilience takes place at different levels—from the level of the individual, across the level of family and school, to the level of societal organisations and policy—which are interconnected and embedded in each other [17]. All these levels have the ability to provide protection. For children, positive relationships with parents lead them to having an important protective system in place and operating in cases of adversity. However, all levels may also be affected by risk: if the parents’ ability to provide a protective context is impaired because of other disadvantageous life circumstances, such as divorce, economic difficulties, or disease, it may build a less strong foundation for the child. This is where protective factors outside the context of the family, such as those related to the school, peers, and spare time, could play a key role for resilient adaptation [18]. Positive experiences within these key contexts would arguably provide children with the sense of having a secure base from which they explore the world, a sufficiently strong notion of self-esteem and self-worth, as well as promote self-directedness or self-efficacy [19]. However, the importance of such protective factors for the association between childhood adversity and adult health needs to be further examined in empirical studies [20].

### 1.3. Study Aim

Children who grow up in families burdened by disadvantage, e.g., unemployment, poverty, or poor health, are sometimes referred to as an at-risk population or a vulnerable population [21]. Gaining a better understanding about risk, protection, and resilience is important not only for the sake of science but for policy aimed at improving the life chances of at-risk populations [10]. Adopting a resilience perspective does not mean that we should accept that these children fare badly at home but, rather, that efforts to reduce risk factors may be complemented by efforts to promote protective factors in other settings as well as at other ecological levels compared to what has traditionally been the case [22]. Hence, variable-based approaches to resilience are not only important to test hypothesized protective factors but can serve as models of intervention [23].

Against this backdrop, the aim of the current study is to explore factors outside the context of the family with the potential to counteract the long-term negative influences of social and material adversity in adolescence on general health status in midlife. The factors were chosen to broadly reflect advantageous conditions with regard to the school, peers, and spare time, hereafter referred to as “protective factors”. The research questions are as follows:

1. Is social and material adversity in youth is associated with poorer self-rated health in midlife?
2. Are protective factors present to a lesser extent among adolescents with experience of adversity?
3. Is the absence of protective factors associated with poorer self-rated health in midlife?
4. Do the protective factors modify the association between social and material adversity in youth and self-rated health in midlife?

## 2. Materials and Methods

### 2.1. Population

The data used was the Northern Swedish Cohort, defined as all individuals who attended the last year of compulsory school (age 16) in 1981, in schools located in the municipality of Luleå (*n* = 1083) [24]. Information about the study was provided to all students and their parents, and only three students declined to participate. The cohort has been surveyed on multiple occasions (1981, 1983, 1986, 1995, and 2008) of which the questionnaire data from 1981 (age 16; Time 1, T1) and 2008 (age 43; Time 2, T2) are employed in the current study. In 1981, data were collected by means of self-administered questionnaires completed during school hours. At approximately the same time, the cohort members’ head teachers were interviewed using a structured interview guide that included questions about each student’s school situation. In 2008, all participants were invited to reunions with the former classmates and asked to complete a questionnaire at the occasion. Those who could not attend the reunion received a postal questionnaire. The study has received approval from the ethical boards at Uppsala University and Umeå University, as well as from the Regional Ethics Vetting Board in Umeå.

### 2.2. Variables

The measure of “Self-rated health” at T2 was based on the question “How would you assess your general health status?” The response options were “Good”, “Poor”, “Somewhere in between”. For the analysis, it was coded so that higher values indicated poorer health.

Derived from the questionnaire at T1, six types of family-related circumstances were used to calculate an index of “Social and material adversity”: parental loss, residential instability, parental illness, poor material standard of living, residential crowding, and parental unemployment. Each of these items were dichotomised so that the value 1 indicated the presence of the adverse condition whereas the value 0 did not. The operationalisation of these items has been described elsewhere [25,26]. The items were subsequently summed up, forming an index ranging from 0 to 6.

The study included 11 measures that reflected an advantageous situation with regard to contexts outside that of the family, e.g., the school, peers, and spare time. Cut offs were chosen to identify the 25% of individuals who were best off in terms of each specific indicator. Due to vast differences in the distributions of the categorical variables, however, this goal was not accomplished for all indicators. The first four indicators were derived from the class teacher interviews at T1: “Educational prospects”, “Work prospects”, “Peer popularity”, and “Scholastic ability”. Of these, two were based on the queries “Try to assess the student’s prospects regarding future studies” and “Try to assess the student’s prospects regarding the labour market”. The response option “Very good” was coded as 1, whereas all remaining options (ranging from “Good” to “Very poor”) were coded as 0. Next, “Peer popularity” was derived from a scale ranging from 1 to 6, where the lowest value reflected non-popularity among peers and the highest value represented popularity among peers. The value 6 was recoded as 1, whereas the remaining values were coded as 0. The indicator of “Scholastic ability” was based on the assessment of the student’s general ability to perform at school. The value “Very high” was coded as 1, whereas all remaining options (ranging from “High” to “Very low”) were coded as 0. The fifth indicator was “School marks”, which was derived from register data on average school marks in the ninth grade (age 16). Values above the 75th percentile were coded as 1 and the remaining values as 0. From the questionnaire distributed to the cohort members at T1, five more items were derived. The first three were “Enjoyment of lessons”, “Enjoyment of breaks”, and “Enjoyment of classmates”, where the response option “Very much” was coded as 1 and the remaining options (ranging from “Quite much” to “Not at all”) were coded as 0. The item “Association/club membership” was derived from the question “Are you a member of any association/club?” The student could tick multiple options, e.g., sports club, sobriety club, scout association, religious association, political association, music association/choir/orchestra, student council, hobby groups, or other associations/clubs. Those who had ticked at least one of these options were coded as 1, whereas the rest were coded as 0. The last item reflected “Quality of spare time” and was based on the question “Is your spare time meaningful to you?” The response option “Yes, to a high extent” was coded as 1, whereas the remaining options (ranging from “Yes, to some extent” to “No, not at all”) were coded as 0. Apart from the ten separate factors specified above, a variable with the summative score was calculated, referred to as the “Protective index”.

Control variables were gender and health at T1. Gender had the value 0 for “Man” and 1 for “Woman”. Since the questionnaire at T1 did not include a question about self-rated health, we used two summary indices reflecting health status. The first was “Internalising symptoms”, consisting of three items reflecting worry/anxiousness, anxiety/panic, and feeling sad/low. The second was “Functional somatic symptoms”, constructed from ten items of physical symptoms including headache, stomach ache, nausea, backache, fatigue, breathlessness, dizziness, overstrain, palpitations, and sleeping difficulties. Details of these indices have been reported in a previous study [27]. In the current study, the measures of internalising symptoms and functional somatic symptoms correlated at 0.53.

As evident in Table 1, the effective sample size is 908 individuals, corresponding to 90% of the individuals participating at both T1 and T2. Nearly all the missing values are due to two of the items included in the index of “Social and material adversity”: parental illness and parental unemployment. For these items, a relatively high percentage of the cohort members answered that they did not know whether or not their father or mother were healthy and/or gainfully employed. Table 1 also presents the distribution of the study variables. Approximately half (48.8%) of the sample are women and the mean scores for internalising symptoms and functional somatic symptoms are overall relatively low (1.1 and 3.6, respectively). Moreover, the mean for “Social and material adversity” is 1.2, suggesting that the sample overall has experience of roughly one type of adverse condition in youth. While not shown in the table, it can be noted that the six types of adversity that formed this index had the following prevalence: parental divorce, separation, or death: 20.0%; residential instability: 18.9%; parental illness: 31.2%; poor material standard of living: 29.0%; residential crowding: 8.0%; and parental unemployment: 11.9%. With regard to the protective factors, our ambition was to identify the top 25% for each factor (the individuals who were the most advantaged). This goal was successfully met for “Educational prospects”, “Work prospects”, “Scholastic ability”, “School marks”, “Association/club membership”, and “Quality of spare time”. However, roughly half of that percentage is noted for high “Peer popularity”, whereas “Enjoyment of lessons/breaks/classmates” is present in 35.6–56.8% of the sample. The mean value of the “Protective index” is 3.0. Concerning self-rated health in midlife, two-thirds have good health whereas only 4.2% report poor health.

### 2.3. Statistical Analysis 

Between T1 and T2, 6.7% of the original cohort were lost to follow-up (including 12 cases of premature death) [24]. Of the remaining 1010 participants, 908 had full information on all variables examined in the current study and were thus included in the analyses. There is an overrepresentation of girls as well as of individuals of higher socioeconomic position among the responders.

**Figure 1:**
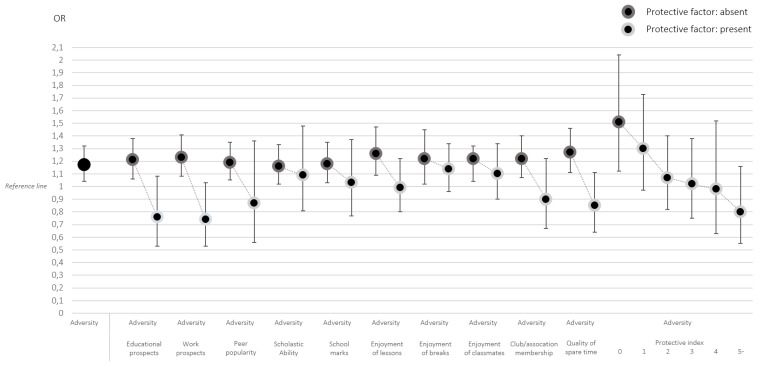
Associations between social and material adversity (ranging between 0 and 6) at T1 (age 16) and self-rated health at T2 (age 43), stratified by (the absence or presence of) each separate protective factor at T1. Results from ordinal regression analysis, presented as odds ratios with 95% confidence intervals (*n* = 908). Adjusted for gender, internalising symptoms, and functional somatic symptoms at baseline. The outcome, self-rated health (ranging between 1 and 3), is coded so that higher vales indicate poorer health.

The analyses were performed in three steps using Stata 15. First, we examined the associations between social and material adversity and the hypothesised protective factors at T1 (Table 2). Since the dependent variables were dichotomous, binomial regression analysis was used. The only exception was for the analysis using the continuous “Protective index” as an outcome, where Poisson regression was applied (producing risk ratios, RRs). The log-link function was applied to handle the high prevalence of the outcomes. The estimates are risk ratios with 95% confidence intervals (CIs). Second, self-rated health at T2 was modelled against each separate protective factor at T1 (Table 3). Ordinal regression analysis was used since this outcome has three hierarchically ordered categories (a test of the proportional odds assumption using the model command showed that this assumption was not violated). This part of the analysis produced odds ratios (ORs) with 95% confidence intervals. Third, the association between social and material adversity at T1 and self-rated health at T2 was analysed, stratified on each separate protective factor at T1 (Figure 1). Ordinal regression analysis was applied here as well. The moderating role of the protective factors was additionally explored through interaction analysis and the results are reported as effect estimates (odds ratios) and *p*-values (Table 4). At each of the steps described above, analyses were adjusted for gender as well as internalising symptoms and functional somatic symptoms at T1. It should be highlighted that these adjustments did not significantly alter the main results. Moreover, interaction analyses were performed to test for gender differences but no significant interactions terms were found.

## 3. Results

Table 2 reports the associations between social and material adversity and the protective factors at T1, based on log-binomial regression analyses with adjustment for gender, internalising symptoms, and functional somatic symptoms at baseline. The association is near null and non-significant for “Enjoyment of lessons/breaks/classmates” as well as “Quality of spare time” but robust and significant for the remaining factors. For example, for every additional adversity, the chance of being assessed by one’s teacher as having very good educational prospects decreases by RR = 0.77 (95% CI = 0.68–0.87). In a similar manner, the likelihood being of member of an association/club decreases by RR = 0.85 (95% CI = 0.76–0.95).

In Table 3, the associations between protective factors at T1 and self-rated health at T2 are presented. The results from ordinal regression analysis suggest that all protective factors are significantly associated with lower odds of having poorer self-rated health (the only exception being “Quality of spare time”). For example, being popular among peers is associated with a lower risk of poorer self-rated health, corresponding to an OR of 0.62 (95% CI = 0.40–0.95).

Figure 1 illustrates the associations between social and material adversity at T1 and self-rated health at T2. The estimates are odds ratios derived from ordinal regression analysis. The leftmost estimate shows that for every additional value increase in adversity, the odds of poorer self-rated health increase (OR = 1.17, 95% CI = 1.04–1.32). The rest of the estimates in the figure show this association stratified by each protective factor. Overall, the results suggest that for the sub samples where the factor is absent, social and material adversity is clearly associated with increased risk of poorer self-rated health. For the sub samples where the factor is present, adversity is not linked to any substantial excess risk of reporting poorer health in midlife. For example, among individuals who report less than “very much” enjoyment of lessons in school, every additional adversity in youth shows an OR of 1.26 (95% CI = 1.09–1.47) for poorer subsequent health. The corresponding OR among those who enjoy lessons very much is 0.99 (95% CI = 0.80–1.22). The part of the figure most to the right shows how the association between adversity and self-rated health stratified according to the “Protective index” (with a score of five or more collapsed into one category). There seem to be certain thresholds: for individuals who do not have any of the ten protective factors, the OR for the association between adversity and health is 1.51 (95% CI = 1.12–2.04). This is the only group for which the confidence interval does not cross the reference line. Then there is a steep decrease in the estimate for those who have one and two factors, respectively (OR = 1.30, 95% CI = 0.97–1.73 and OR = 1.07, 95% CI = 0.82–1.40). The estimates for those with three and four factors remain roughly at the same level (OR = 1.02, 95% CI = 0.75–1.38 and OR = 0.98, 95% CI = 0.63–1.52) whereas a larger decrease again can be noted for those with five or more factors (OR = 0.80, 95% CI = 0.55–1.16).

The stratified analyses are formally tested through an interaction analysis, shown in Table 4. The interaction term between social and material adversity and the “Protective index” as a whole is statistically significant. Looking at the separate factors, all interaction terms point in the same direction but only three of them are statistically significant at the 95% level: “Educational prospects”, “Work prospects”, and “Quality of spare time”.

## 4. Discussion

The aim of the current study was to explore factors outside the context of the family with the potential to counteract the long-term negative influences of social and material adversity in adolescence on general health status. We will now discuss the results of the study, structured according to the four research questions formulated earlier.

### 4.1. Disadvantaged Youth Have Poorer Health as Adults

First, we asked whether social and material adversity in youth would be associated with poorer self-rated health in midlife. Our results support that notion. This is also in line with several other studies based on the Northern Swedish Cohort [25,26]. Among the factors included in the summary index of adversity, sensitivity analyses (data not presented) showed that the following three items contributed the most to this association: parental illness, residential crowding, and parental unemployment. Due to strong clustering of adversities in youth and their multiplicative effects on health, however, caution should be taken in interpreting influences from single adversities [28]. The current study did not investigate any intermediate factors linking youth adversity to adult health.

Nevertheless, drawing inspiration from cumulative inequality theory, we presume that disadvantages in multiple life domains in childhood and adolescence may hamper the individual’s health development both directly and through the exposure to subsequent risk factors that in turn have negative health consequences. The notion of cumulative life-course processes that encompasses a multidimensional approach to social, economic, and health-related factors has received empirical support in other cohort studies [25,29,30,31,32,33]. 

### 4.2. Disadvantage within the Family Is Linked to Disadvantage Outside the Family

Second, our findings suggest that the protective factors are less present among adolescents with experience of adversity. When looking at the summative index of protective factors in youth, the regression analysis showed that for every additional type of adversity there was a statistically significant decrease in the number of protective factors. We may exemplify this by the fact that only 8% of the adolescents coming from a strongly adverse background (i.e., three or more types of adverse conditions) had five or more protective factors, whereas the corresponding percentage among adolescents without any on the studied adverse living conditions was 31%. Focusing on each separate factor, however, the results suggested that adversity was not significantly related to the three items reflecting the enjoyment of lessons, breaks, and classmates, or to the quality of spare time. The former finding can be due to the relatively high prevalence of strong enjoyment—it was uncommon in this sample to not report enjoyment at all—which could suggest that these indicators (when reversed) may work better as risk factors. The latter finding is more difficult to interpret. Hypothetically, it is possible that adolescents who face difficulties at home feel more at ease outside the context of the family and therefore might see their spare time as more meaningful. As a consequence, the differences in quality of spare time between them and peers who do not experience social and material adversity would be mitigated. Adversity showed statistically significant negative associations with the six remaining factors: future prospects regarding education and work, popularity among peers, school marks, and being member of an association/club. This is in line with past research [18].

### 4.3. An Advantageous Situation Outside the Family Is Related to Better Health Development

Third, adolescents for whom protective factors were present were less likely to rate their health as poorer in midlife. This was reflected by the fact that the “Protective index” showed decreased odds of poorer self-rated health in midlife. With the exception for the item reflecting the cohort members’ quality of spare time, all specific protective factors showed negative associations with poorer self-rated health. The association with health was slightly stronger for the factors assessed by the teachers compared to the items from the student questionnaire. All in all, the associations found here correspond well to our previous studies focusing on future prospects, scholastic ability and school marks [34], popularity among peers [35], and membership in associations or clubs [36], in relation to health development.

### 4.4. Factors Outside the Family Protect Against the Poor Health Stemming from Youth Adversity

Fourth, the results showed that the association between adversity in youth and health in midlife was moderated by most of our investigated factors related to school, peers, and spare time. More specifically: among individuals for whom the studied protective factors were absent, the association between adversity and midlife health was robust and statistically significant, whereas it was weak and in many cases statistically non-significant among individuals for whom the factors were present. These findings were formally tested through interaction analysis, where three factors were found to significantly interact with social and material adversity in their influence on subsequent health: “Educational prospects”, “Work prospects”, and “Quality of spare time”. Here, it is necessary to reflect upon what these factors really measure. The former two were based on the class teachers’ assessments and were most likely guided by rather holistic judgements of the students’ competences and performance at school. For example, correlation analysis revealed that these two measures were quite strongly correlated with “Scholastic ability” (*r* = 0.66 and *r* = 0.55, respectively) and “School marks” (*r* = 0.56 and 0.47, respectively), as well as to each another (*r* = 0.77). Many studies have nevertheless shown, even after adjusting for previous academic achievements, that young people actually achieve higher levels of academic success if teachers see them as capable and expect them to perform well [37]. The indicators reflecting educational and work prospects may thus not only reflect academic success but also positive relationships with teachers and other school personnel, factors that we know are particularly important for the outcomes of children coming from adverse backgrounds [38]. The similar complexity applies to the students’ assessment of the “Quality of spare time”. Here, we need to reflect upon what a meaningful spare time means. In the questionnaire, this item is accompanied by the proposition to think about whether one learns something new and develop as a person during one’s spare time. The respondents may nevertheless have considered a wide range of aspects related to the specific activities taking place during their spare time. For example, this item was only weakly correlated with “Association/club membership” (*r* = 0.24). Previous research, based on youths living in the industrialized parts of the world, has considered five areas of spare time activities that may be meaningful [19]: cultural pursuits (e.g., performing dance or singing in a choir), care of animals, sports, helping and volunteering (participation in community service, e.g., peer tutoring), and part-time work. There are several reasons why such activities would protect against the negative health consequences of childhood adversity: they might for example help the child to develop instrumental and social skills, strengthen social networks, enhance sense of self-esteem and self-efficacy, increase physical fitness, and promote a sense of belonging and purpose in daily living [19].

### 4.5. Methodological Strengths and Limitations

Major strengths of the current study were the longitudinal design, prospective data collections, large sample size, and very small loss to follow-up. Moreover, it was possible to include multiple indicators that captured relatively objective aspects of social and material adversity, as well as protective factors related to the school, peers, and spare time of which some were assessed by the class teachers. Using self-rated health in adulthood as the outcome of interest provided a reliable indicator of general health status that was less likely to overlap with the concept of resilience as compared to mental health indicators. Some limitations of the study should nevertheless be highlighted. Most importantly, while we acknowledge that one of the key assumptions of our theoretical framework is that the human life courses are dynamic processes, this was not directly addressed by the empirical analyses. Only two measurement points were used: age 16 and age 43. Future studies should additionally examine risk and protective factors at the other available time points (ages 18, 21, and 30) to better capture stability and change across the life course. Furthermore, although we are able to control for functional somatic symptoms at baseline, there could still be unmeasured confounding. Another limitation concerns our measurement of social and material adversity which is relatively crude. For instance, we do not know what kind of illness the parents were suffering from or how long the parents had been unemployed. It is possible that the protective factors in fact reflect the degree of severity of the adverse conditions, in the sense that the presence of one or more protective factors could be the result of an environment that is in fact not as adverse as it may appear. In a similar vein, rather than being protective factors, aspects such as scholastic ability and school marks could act as mediators between adversity and health in midlife, and could thus reflect successful resilience rather than being the causes of resilience. This is nevertheless something that will be difficult to disentangle using observational data.

## 5. Conclusions

To conclude, the present study found that social and material adversity in youth was associated with poorer health in midlife. The results also indicated that protective factors related to peers, school, and spare time, were present to a lesser extent among adolescents with experience of adversity and that their absence was associated with poorer subsequent health. Finally, the association between youth adversity and adult health was weaker among those for whom protective factors were present. Although resilience in this study was defined as the extraordinary capacity of human beings to prevail over adversity, the protective factors we found to be the most influential—as reflected through educational prospects, work prospects, and quality of spare time—do not require fostering of any superhero skills, just some “ordinary magic” [23]. This is a fact that holds promise both for practical reasons as well as for social and health policy. Future studies should further examine the ways in which positive future prospects and high quality of spare time crystallise across youth.

## Figures and Tables

**Table 1:** Descriptive statistics for all study variables (*n* = 908).

Study Variables	*n*	%
*Main dependent variable* (T2)		
Self-rated health		
Good	596	65.6
Intermediate	274	30.2
Poor	38	4.2
*Main independent variable* (T1)		
Social and material adversity	Mean = 1.2, S.D. = 1.1, Range = 0–5	
*Protective factors*^a^ (T1)		
Educational prospects	219	24.1
Work prospects	236	26.0
Peer popularity	122	13.4
Scholastic ability	208	22.9
School marks	248	27.3
Enjoyment of lessons	379	41.7
Enjoyment of breaks	516	56.8
Enjoyment of classmates	324	35.7
Association/club membership	227	25.0
Quality of spare time	229	25.2
Protective index	Mean = 3.0, S.D. = 2.3, Range = 0–10	
*Control variables* (T1)		
Gender (woman)	443	48.8
Internalising symptoms	Mean = 1.1, S.D. = 1.3, Range = 0–8	
Functional somatic symptoms	Mean = 3.6, S.D. = 2.7, Range = 0–16	

T1 = Time 1, age 16; T2 = Time 2, age 43. ^a^ The frequency and percentage distribution presented here reflect the most advantageous situation.

**Table 2:** Associations between social and material adversity and the protective factors at T1. Results from log-binomial regression analyses, presented as odds ratios per one-unit increase in the measure of social and material adversity (*n* = 908). Statistically significant (*p* < 0.05) estimates in bold. Adjusted for gender, internalising symptoms, and functional somatic symptoms at baseline. CI = confidence interval; OR = odds ratio.

Dependent Variables:	Independent Variable: Social and Material Adversity	
	OR	95% CI
Educational prospects ^a^	**0.77**	0.68–0.87
Work prospects ^a^	**0.78**	0.70–0.88
Peer popularity ^a^	**0.79**	0.67–0.94
Scholastic ability ^a^	**0.83**	0.74–0.94
School marks ^a^	**0.77**	0.69–0.85
Enjoyment of lessons ^a^	0.97	0.90–1.04
Enjoyment of breaks ^a^	1.00	0.95–1.05
Enjoyment of classmates ^a^	0.97	0.89–1.05
Association/club membership ^a^	**0.85**	0.76–0.95
Quality of spare time ^a^	0.95	0.86–1.06
Protective index ^b^	**0.89**	0.86–0.92

T1 = Time 1, age 16. ^a^ Coded so that the value 1 reflects the most advantageous situation, whereas the value 0 indicates a less advantageous situation. ^b^ Assessed with Poisson regression analysis, producing incidence—rate ratios.

**Table 3:** Associations between the protective factors (separate model for each factor) at T1 and self-rated health at T2. Results from ordinal regression analyses presented as odds ratios (*n* = 908). Statistically significant (*p* < 0.05) estimates in bold. Adjusted for gender, internalising symptoms, and functional somatic symptoms at baseline.

Independent Variables: ^a^	Dependent Variable: Self-Rated Health ^b^	
	OR	95% CI
Educational prospects	**0.54**	0.38–0.76
Work prospects	**0.56**	0.40–0.78
Peer popularity	**0.62**	0.40–0.95
Scholastic ability	**0.60**	0.43–0.85
School marks	**0.61**	0.44–0.84
Enjoyment of lessons	**0.65**	0.49–0.86
Enjoyment of breaks	**0.76**	0.57–1.00
Enjoyment of classmates	**0.72**	0.53–0.96
Association/club membership	**0.70**	0.51–0.98
Quality of spare time	1.01	0.74–1.38
Protective index	**0.86**	0.80–0.91

T1 = Time 1, age 16; T2 = Time 2, age 43. ^a^ Coded so that the value 1 reflects the most advantageous situation, whereas the value 0 indicates a less advantageous situation. ^b^ Coded so that higher values indicate poorer health.

**Table 4:** Interactions between social and material adversity and the protective factors at T1 (age 16) in their effect on self-rated health at T2 (age 43). Results from ordinal regression analysis, presented as odds ratios with *p*-values (*n* = 908). Adjusted for gender, internalising symptoms, and functional somatic symptoms at baseline.

Interactions Terms: ^a^	Dependent Variable: Self-Rated Health ^b^	
	OR	95% CI
Adversity × Educational prospects	0.67	0.46–0.96
Adversity × Work prospects	0.66	0.47–0.93
Adversity × Peer popularity	0.74	0.47–1.14
Adversity × Scholastic ability	0.95	0.69–1.30
Adversity × School marks	0.90	0.66–1.22
Adversity × Enjoyment of lessons	0.79	0.61–1.02
Adversity × Enjoyment of breaks	0.91	0.72–1.16
Adversity × Enjoyment of classmates	0.87	0.68–1.11
Adversity × Association/club membership	0.76	0.55–1.05
Adversity × Quality of spare time	0.72	0.54–0.96
Adversity × Protective index	0.87	0.85–0.96

T1 = Time 1, age 16; T2 = Time 2, age 43. ^a^ Each interaction term is entered in a separate model together with the two main terms. ^b^ Coded so that higher values indicate poorer health.
